# Physical Properties of Blood and their Relationship to Clinical Conditions

**DOI:** 10.3389/fphys.2022.906768

**Published:** 2022-07-06

**Authors:** Tamas Alexy, Jon Detterich, Philippe Connes, Kalman Toth, Elie Nader, Peter Kenyeres, Jose Arriola-Montenegro, Pinar Ulker, Michael J. Simmonds

**Affiliations:** ^1^ Department of Medicine, Division of Cardiology, University of Minnesota, Minneapolis, MN, United States; ^2^ Department of Pediatrics, Division of Cardiology, Children’s Hospital Los Angeles, Keck School of Medicine, University of Southern California, Los Angeles, CA, United States; ^3^ Laboratory LIBM EA7424, Vascular Biology and Red Blood Cell Team, University of Lyon, Lyon, France; ^4^ First Department of Medicine, Division of Cardiology, Medical School, University of Pecs, Pecs, Hungary; ^5^ Department of Physiology, Akdeniz University, Faculty of Medicine, Antalya, Turkey; ^6^ Biorheology Research Laboratory, Menzies Health Institute Queensland, Griffith University, Brisbane, QLD, Australia

**Keywords:** blood viscosity, hemorheology, medical devices, aggregation, deformability

## Abstract

It has been long known that blood health heavily influences optimal physiological function. Abnormalities affecting the physical properties of blood have been implicated in the pathogenesis of various disorders, although the exact mechanistic links between hemorheology and clinical disease manifestations remain poorly understood. Often overlooked in current medical practice, perhaps due to the promises offered in the molecular and genetic era, the physical properties of blood which remain a valuable and definitive indicator of circulatory health and disease. Bridging this gap, the current manuscript provides an introduction to hemorheology. It reviews the properties that dictate bulk and microcirculatory flow by systematically dissecting the biomechanics that determine the non-Newtonian behavior of blood. Specifically, the impact of hematocrit, the mechanical properties and tendency of red blood cells to aggregate, and various plasma factors on blood viscosity will be examined. Subsequently, the manner in which the physical properties of blood influence hemodynamics in health and disease is discussed. Special attention is given to disorders such as sickle cell disease, emphasizing the clinical impact of severely abnormal blood rheology. This review expands into concepts that are highly topical; the relation between mechanical stress and intracellular homeostasis is examined through a contemporary cell-signaling lens. Indeed, accumulating evidence demonstrates that nitric oxide is not only transported by erythrocytes, but is locally produced by mechanically-sensitive enzymes, which appears to have intracellular and potentially extracellular effects. Finally, given the importance of shear forces in the developing field of mechanical circulatory support, we review the role of blood rheology in temporary and durable mechanical circulatory support devices, an increasingly utilized method of life support. This review thus provides a comprehensive overview for interested trainees, scientists, and clinicians.

## Introduction

The physical properties of blood have been under examination for millennia, ancient Greek investigators understood the vital essence of blood and its role in disease development (Meletis and Konstantopoulos, 2010). The advent of optical microscopy facilitated further understanding of the complexity of blood. Scientists, such as Van Leeuwenhoek, observed the sedimentation of red blood cells (RBC) and their ability to deform in order to traverse capillaries smaller than themselves (Martins e Silva, 2009). The monumental discoveries that contributed to the study of fluids can be attributed to the work of Jean-Leonard-Marie Poiseuille, Gotthilf Heinrich Ludwig Hagen, Claude-Louis Navier, and George Stokes. Independently, they established the fundamental relationships between flow rate, pressure differential, vessel dimensions, and the viscosity of the fluid, deriving the equations that now bear their name. Concerning blood viscosity, it was their inability to apply their approaches to blood that indirectly demonstrated its non-Newtonian properties.

Blood is the archetype of a non-Newtonian fluid, defined as a fluid with a shear rate dependent viscosity. Specifically, blood is shear-thinning, whereby the viscosity decreases as shear rate increases. In this document, we aim to review the factors that determine the physical properties of blood and their alterations in various clinical conditions. More specifically, we focus on the following areas: *Non-Newtonian properties of blood; Determinants of blood viscosity; Hemodynamic impact of various hemorheological parameters; Contribution of altered hemorheological parameters to the pathogenesis of various diseases; Novel concepts and trends in contemporary blood rheology, and the Role of hemorheology in temporary and durable mechanical circulatory support devices.*

### Non-Newtonian Behavior Concept and the Definition of Blood Viscosity

External forces acting on a fluid promote flow while its internal friction forces impede it. The magnitude of the external force acting parallel to the direction of displacement (termed shear stress) is directly proportional to the rate of change of deformation in the material (e.g., fluid). Shear stress is particularly relevant in the circulatory system as it has profound effects on both blood cells and the vascular wall. The related property, shear rate, is dependent on the velocity gradient that exists between the slower flowing regions near the endothelium and the faster regions at the axial center of the vessel. Blood viscosity is derived from the shear rate-shear stress relationship. Fluids with higher viscosity exhibit less deformation and slower flow compared with lower viscosity fluids; and higher viscosity fluids require higher external force to maintain flow. Viscosity depends on several factors intrinsic to the fluid, such as temperature, pressure, density, and composition. It also depends on the amplitude and frequency of external forces to which it is exposed. In “*Newtonian*” fluids, such as water and plasma, shear stress exhibits a linear relationship with shear rate, therefore, viscosity is constant and independent of shear rate. Plasma, a solution of electrolytes, proteins and other macromolecules, is a prototypical Newtonian fluid but is only one component of whole blood.

**FIGURE 1:**
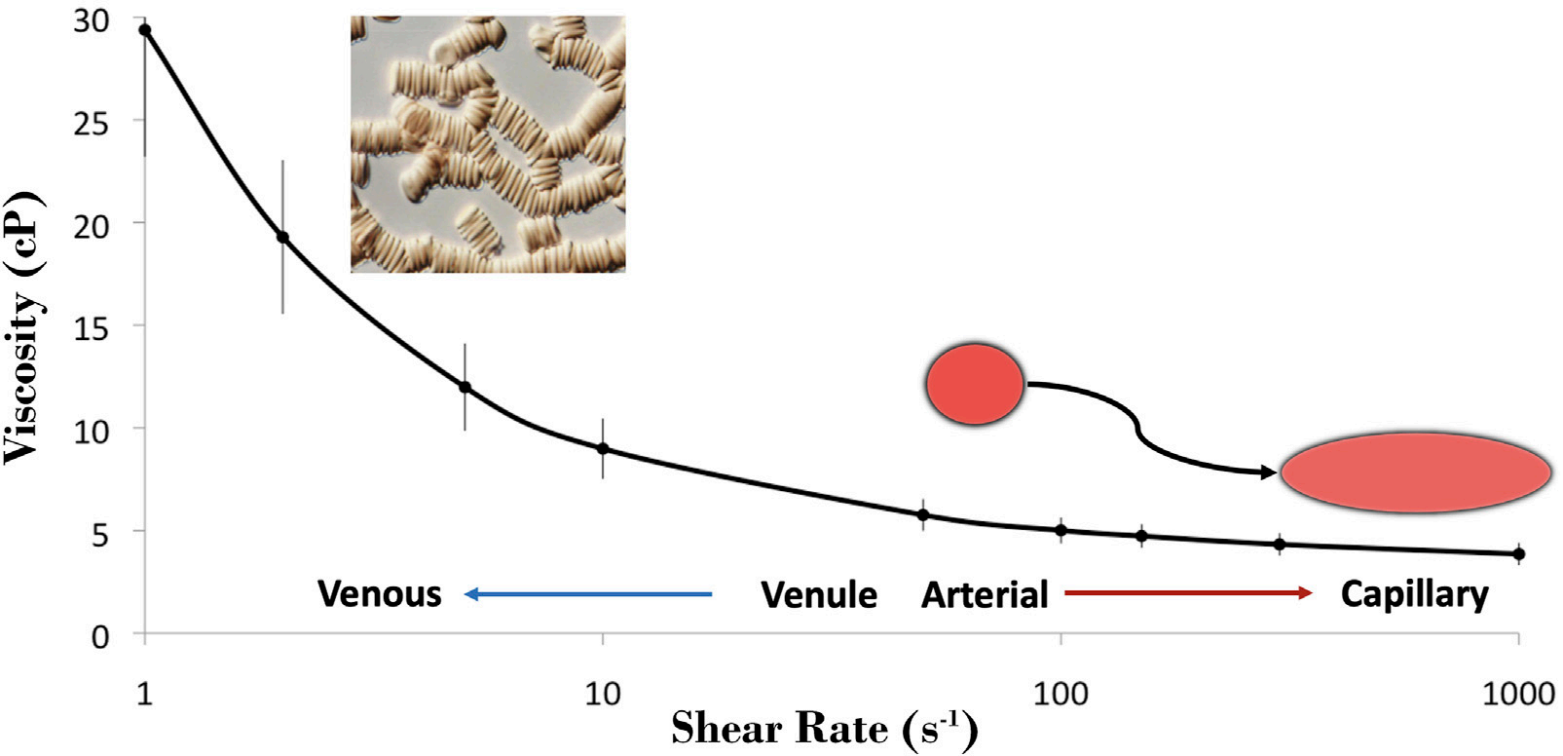
Blood is a shear-thinning fluid, where it is most thick within the venous network with lower shear and it becomes progressively thinner under the higher shear regions of the arterial network and microcirculation. The tendency for RBC to cluster (i.e., aggregate; micrograph) is the primary determinant of blood viscosity at low shear, and the dispersion of these clusters aids in dramatically decreasing blood viscosity with increasing shear. The shear thinning observed at >100 s^−1^ may be attributed to the unique capacity of RBC to deform from their resting discocyte shape to hydrodynamically favorable morphologies, thus reducing the internal resistance of blood.

Whole blood is a complex fluid suspension of cellular elements in plasma that results in a non-linear relationship between shear rate and shear stress. The viscosity of whole blood demonstrates a non-linear decrease with increasing shear rate, described as shear thinning behavior (Figure 1). RBC aggregation is the primary determinant of blood viscosity under low shear conditions. Rising shear forces break up three dimensional rouleaux and the importance of individual erythrocyte deformability rises to prominence. Artificially eliminating these phenomena by “rigidifying” RBC and suspending them in a non-aggregating medium turns the solution into a fluid with essentially Newtonian behavior. Furthermore, above a certain shear rate, the change in viscosity of whole blood becomes linear and eventually flattens, mimicking a Newtonian fluid.

*In vivo*, the shear rate is zero at the center of the flow column and increases progressively towards the vascular wall. This yields an indefinite number of varying local viscosity values across the radius of the vessel. In order to simplify blood flow calculations, the continuum model of blood flow is used, where flow of blood is described by its bulk movement and the term “apparent viscosity” is used. It is determined by the flow rate achieved in response to the pressure differential in the circulation and the flow geometry, all of which are defined by the Hagen-Poiseuille equation. As vessel geometry decreases progressively towards the microcirculation, their diameter ultimately approaches the size of individual blood cells and the applicability of predictions made using bulk-flow calculations no longer apply. Flow observed at this scale (e.g., <200–300 μm vessel diameter) promotes “phase separation” of blood. This results from the more deformable RBC migrating to the center of the flow axis thus developing a cell-rich, higher local viscosity inner core and a cell-poor, plasma-rich low viscosity external layer. The lower viscosity of the outer plasma phase facilitates higher shear rate flow along the vascular endothelium, thereby reducing resistance to flow. Important concepts that explain this phenomenon, for the curious reader, are mechanistically explained by the: 1) Fåhraeus effect, which describes why the local hematocrit within a region of the microcirculation is lower than expected due largely to faster velocity of RBC at these scales than the bulk fluid, and 2) Fåhraeus-Lindquist effect, which explains that the ‘apparent viscosity’ is lower than expected as a result of phase separation. At vessel bifurcations, phase separation leads to unequal partition of plasma and cells to the *daughter* branches based on their angle of origin and diameter, resulting in inhomogeneous hematocrit among small vessels. In the smallest capillaries, cells may squeeze through in single-file flow, and their deformability and internal viscosity becomes the predominant characteristic instead of bulk flow properties.

## Determinants of Whole Blood Viscosity

Several factors influence whole blood viscosity. These include characteristics of the vascular bed, the whole blood, intrinsic red blood cells properties, and various plasma factors. We will individually review the role of these below.

### Shear Rate in Different Vascular Segments

The non-Newtonian behavior of blood is dependent on the dynamic nature of flow within the circulatory system, and corresponding variance of shear rates in successive vascular compartments of variable radius. Apparent shear rate is proportional to the blood flow velocity, which is highest at the centerline and decreases progressively towards the vessel wall according to the following equation:

γ=Δvh (1)

γ is the shear rate in s^−1^, Δv is the radial gradient of flow velocity within a vessel, and h is the radial distance between the two measured points (by convention, h is often measured as the distance from the vessel wall but a more accurate shear map is made using shorter distances that are based on internal velocity changes).

RBC aggregates may form in large veins where blood velocity is low with minimal gradient change towards the vascular wall (i.e., lower shear rate), promoting a rise in apparent whole blood viscosity (Reinke et al., 1986). The increase in velocity and hemodynamic shear forces as blood travels through the vena cavae and cardiac chambers disperses the rouleaux (Brooks et al., 1970). The high shear forces in the arterial system maintain RBC monodispersion, a phenomenon that ultimately facilitates single-file flow within the microcirculation. While the dependence of whole blood viscosity on shear rate defines bulk blood flow, a systematic approach is required for a thorough examination of how each component affects viscosity over the physiologic range of shear rates.

### Hematocrit

Erythrocytes represent the largest cellular component of blood. Hematocrit, defined as the volume of RBC compared to total blood volume (normal range: 35–45%), is the primary determinant of blood viscosity, especially at the lowest shear rates: doubling of hematocrit results in a 3-to-4-fold increase in blood viscosity at high shear rates (predominantly a mass effect), while it prompts an almost 10-fold increase within the lowest shear rate range (mass effect plus aggregation/rouleaux formation) (Chien et al., 1966).

### Red Blood Cell Aggregation

One of the most common observations made when examining RBC suspensions in plasma under light microscopy is the tendency for RBC to gravitate towards each other, initially into linear columns (resembling stacks of coins, or rouleaux), and gradually into 3-dimensional clusters. This process has been extensively examined in a book by Baskurt and others (Baskurt et al., 2012); interested readers are guided to this valuable resource. Aggregate formation is relatively universal within the mammalian kingdom; species from broad divisions such as monotremes (e.g., echidna), marsupials (e.g., koala), and placental mammals (e.g., humans) all exhibit varying degrees of this phenomenon (Baskurt et al., 2010). The precise mechanisms that define RBC aggregation were intensely studied in the 20th century, allowing a thorough understanding of this controversial process. There are two theorized models of RBC aggregation. Early scholars promoted that RBC aggregates form via fibrinogen bridging, whereby RBC are attracted to one another through attachment to macromolecules, such as fibrinogen, in a process that interlocks cells (Chien and January 1973). This is termed the “bridging model”. Subsequently, exquisite biophysical models, both theoretical and experimental, led to the discovery that fibrinogen did not necessarily cause RBC bridging, but rather, due to its immense size and physical characteristics, attracted fluid via local oncotic pressure gradient (Neu and Meiselman, 2002). As a result, the intercellular distance between adjacent cells is reduced to an extent that RBC align into tight linear rouleaux and eventually branching clusters.

The determinants of RBC aggregation can be categorized into those that are extrinsic to the cell, such as local shear rate, hematocrit, and plasma protein concentration, and those dependent on the intrinsic properties of erythrocytes, including surface charge, and membrane fluidity. In general, *shear rate* is considered a “disaggregating” factor given that rouleaux are only stable at stasis or under a tolerable low shear rate. Aggregates disperse as shear increases, profoundly reducing blood viscosity. *Hematocrit* is “pro-aggregating” because higher RBC concentrations promote cell-cell contact. Elevated *plasma protein concentrations*, and particularly fibrinogen, enhance aggregation. Given the overall negative surface charge of the *RBC membrane*, they tend to repel each other. Elimination of negatively charged glycoproteins from the surface (e.g., sialic acid), will increase RBC aggregation. Finally, it is known that conditions that severely impact *RBC membrane fluidity* lead to decreased RBC aggregation, emphasizing that a certain minimal level of erythrocyte deformability is essential for the process (Baskurt et al., 1998). However, aggregates of poorly deformable RBC tend to be very robust and difficult to disperse with increasing shear rate.

It is important to emphasize that RBC aggregation is not the same as clot formation or agglutination. Aggregation occurs normally in the circulation of healthy humans, thus it should not be regarded as detrimental if within tolerable limits. However, pathological *hyperaggregability* is a non-specific marker in a wide range of diseases, including inflammatory disorders.

### Red Blood Cell Deformability

The viscosity of blood is greatly influenced by the exceptional ability of RBC to adjust their morphology in response to local shear forces (Mauer et al., 2018). Decreased RBC deformability occurs when the membrane lipid bilayer loses fluidity, if the internal viscosity of the cell increases, or when the cell’s size and/or hydration status is impacted, compromising its exceptional surface area-to-volume ratio. Decreased deformability promotes an increase in blood viscosity at high shear rates, owing to the greater impact of individual RBC mechanics in the microcirculation with high shear rate.

When exposed to high shear forces, RBC with normal deformability tend to: 1) orientate with the direction of flow rather than maintaining a random alignment; 2) align on an angle that promotes a streamlined approach to flow; 3) the cell membrane rotates around the cytosol, at least in higher viscosity environments (McNamee et al., 2020). This latter phenomenon has been well-decribed and quantified recently *in vitro* (Recktenwald et al., 2022). *In vivo*, it appears that the cell membrane would not rotate around the cytosol, at least at lower hematocrit regions such as the microcirculation, because the suspending media (i.e., plasma) viscosity is lower than the RBC internal viscosity. Lanotte demonstrated that upon exposure to increasing shear rates and when the viscosity contrast between the cytosol and plasma is large enough, RBC first tumble, then roll, transit to a rolling and tumbling stomatocyte, and finally adopt a polylobed shape (Lanotte et al., 2016). These processes all occur in a relatively predictable relation with shear rate and coincide with the shear-thinning of blood, thus these complex interactions are thought to facilitate reduced blood viscosity under higher shear conditions.

The “deformability” of RBC is unique among mammalian cells and determines the bulk flow properties of blood as well as the capacity of these cells to traverse the microcirculation. Sickle cell disease is a classic clinical example that demonstrates the profound impact of elevated intracellular viscosity (among other factors) on RBC deformability, as discussed in detail below.

### Suspending Medium–Plasma Viscosity and the Effect of Protein Content

Plasma, the suspending medium for the cellular components of blood, plays an important function in moderating the physical properties of blood. The viscosity of plasma is determined principally by its protein content, although with inequivalent contribution: larger proteins with certain physical characteristics exert a greater impact on plasma viscosity. Given that plasma separated for experimental purposes may be contaminated by residual platelets and white blood cells, for this review, we will refer to plasma viscosity as that of pure plasma, completely free of cellular elements.

Plasma is a Newtonian fluid whereby its viscosity is independent of the shear rate it is exposed to. Its normal value at 37°C is around 1.3 mPa s. To put this into perspective, the apparent viscosity of blood at 40% hematocrit and a shear rate of 1,000 s^−1^ is approximately 5 mPa s with 30% plasma contribution. However, if the same blood is exposed to a very low shear rate of 0.1 s^−1^ where blood viscosity is 65 mPa s, plasma contribution would merely account for 2%. Under certain circumstances, such as acute inflammation or select malignancies, plasma viscosity may double or even triple thereby significantly increasing apparent blood viscosity, especially at high rates of shear (Chien et al., 1966). Conversely, the effect of enhanced rouleaux formation owing to a rise in acute phase reactants in the plasma is much more profound at low shear rates. Fibrinogen is an important determinant of plasma viscosity, due to its high molecular weight (∼340 kDa), and *fibrous* structure. Given that fibrinogen is also the primary non-cellular determinant of RBC aggregation as discussed above, this protein impacts blood viscosity through two independent mechanisms. Other circulating acute phase reactants, such as immunoglobulins and C-reactive protein have a smaller impact on plasma viscosity and RBC aggregation. Owing to its ubiquitous nature and high concentration in plasma, albumin also significantly impacts plasma and whole blood viscosity.

## The Hemodynamic Impact of Hemorheological Parameters

In clinical medicine, resistance across the vasculature is of critical importance since it is a key determinant of systemic blood pressure and distribution of blood flow, ultimately determining organ specific oxygen delivery. Resistance to flow is largely dependent on the properties of blood (primarily viscosity) and demonstrates an inverse relationship with the overall vascular cross-sectional area. As providers, we make clinical decisions concerning vasoregulatory interventions on a daily basis after considering vascular resistance. This is of critical importance in the intensive care setting and in the management of patients with advanced heart failure. In pulmonary hypertension, for example, we measure the pressure drop across the pulmonary vascular bed and cardiac output to determine pulmonary vascular resistance according to the following equation:

R=ΔP/Qp (2)

where *R* = resistance, *p* = pressure, and *Qp* = pulmonary artery flow rate.

When direct measurement is not possible, describing pressure drop across a vascular segment using mathematical modeling may be complicated. Jean-Leonard-Marie Poiseuille and Gotthilf Heinrich Ludwig Hagen were the first to identify contributing factors in ideal situations, and with the following assumptions of the fluid: 1) it is non-compressible; 2) it shows Newtonian behavior, and 3) it maintains a laminar flow without acceleration. This led to the definition of the Hagen-Poiseuille equation, which includes viscosity as a key parameter:

$$R=\frac{8\mu L}{\pi r^{4}}\Delta P=\frac{8\mu LQ}{\pi R^{4}}=\frac{8\pi \mu LQ}{A^{2}}$$ (3)

where *μ* = viscosity, *L* = length, *Q* = flow rate*, A* = cross sectional area of flow.

It is important to emphasize the assumption that the fluid is Newtonian, which is not the case for blood. More complex fluid models, such as the Casson fluid model, attempt to account for the non-Newtonian behavior of blood. One of the primary research goals in the field of hemorheology is to determine whether individual rheological parameters can be manipulated in order to favorably impact hemodynamics.

### The Endothelium, Poiseuille-Hagen Principle, and Vascular Tone

Detailed review of the structure and function of the vascular endothelium is beyond the scope of this review. However, it is important to briefly emphasize that the endothelium serves as a physical barrier controlling fluid, solute, and cellular transit from the intravascular compartment to the tissues and intercellular space. It also acts as a mechano-transducer and a chemical sensor to modulate vascular tone. The endothelium senses variations in volumetric blood flow, velocity, viscosity, and releases vasoregulatory signals in response targeting the vascular smooth muscle cell layer (Green et al., 2014). Local vascular resistance and pressure gradients may thus remain under tight regulation, at least in healthy vascular beds. Importantly, vessels denuded of the endothelial layer respond by vasoconstriction (myogenic response) to such rheological challenges (i.e., altered flow, velocity, viscosity) (Bryan et al., 2001). This has important consequences in numerous disorders with damage to the endothelial layer, such as coronary artery disease, sickle cell disease, and certain types of vasculitis.

### RBC Deformability

**FIGURE 2:**
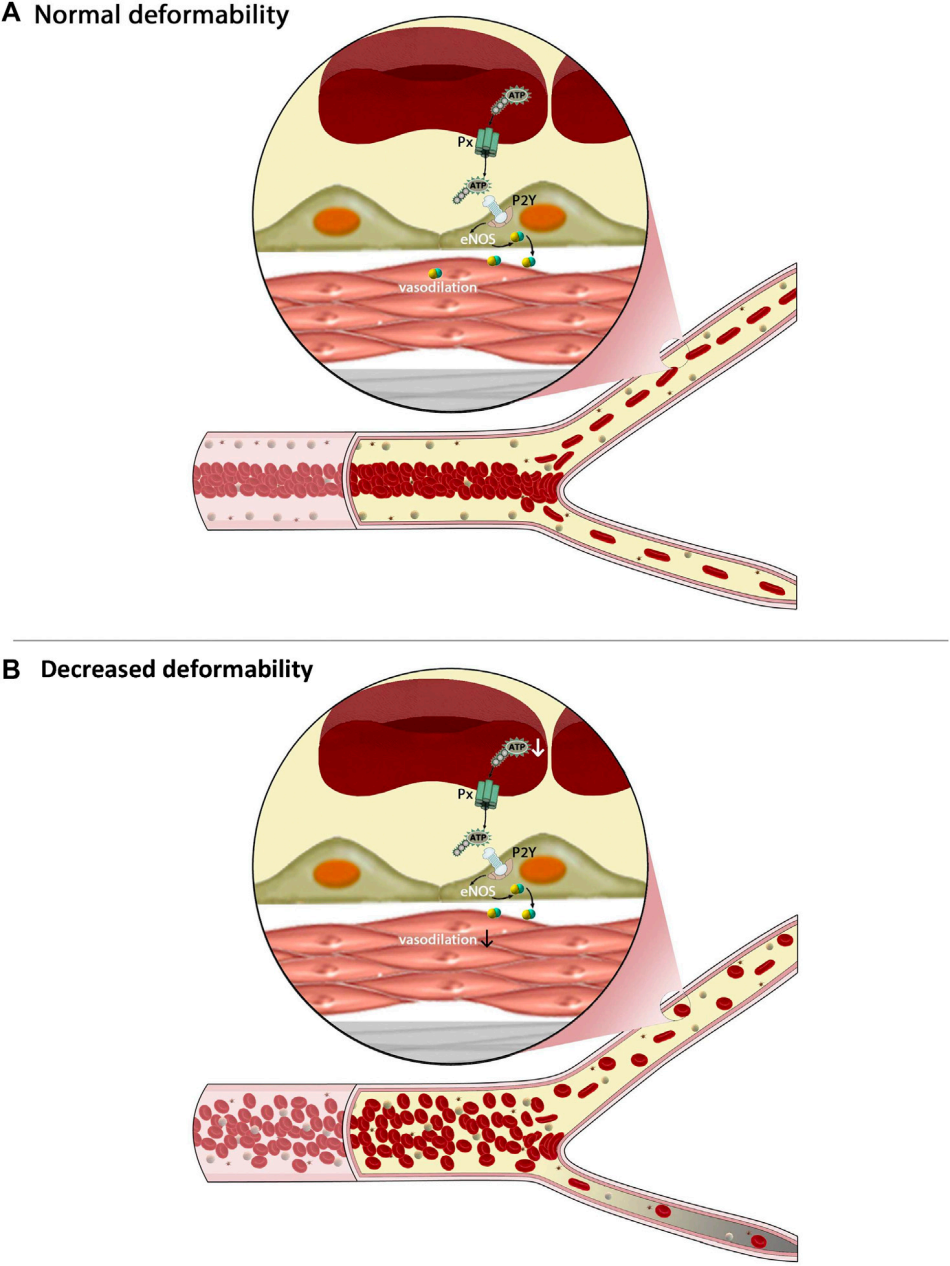
The figure demonstrates some of the complex interactions between cellular mechanics of red blood cells (RBC), the flux of the microcirculation, and intracellular signaling within these cells. Under healthy conditions, RBC are highly deformable, which enables them to migrate to the center of flow thereby creating a cell-poor layer **(A)** and to pass easily throughout daughter branches of the microcirculation. Less deformable RBC **(B)**, however, tend to have greater heterogeneity of distribution within larger vessels, resulting in a much narrower cell-poor layer and thus greater wall shear stress. Upon reaching bifurcations, these rigid RBC are unable to enter smaller capillaries, prompting a maldistribution of RBC and impaired flux of these pathways. Other mechanisms are also of critical importance, such as hypoxic vasodilation, yet not shown on this figure.

Hemodynamic parameters can be affected by altered RBC deformability in multiple ways. As discussed earlier, impaired cellular deformability has a profound impact on blood viscosity and therefore may limit local tissue perfusion and oxygen delivery secondary to suboptimal convective transport (Chien, 1987; Pantely et al., 1988). In addition, upon entry to the microcirculation, rigid RBC exhibit impaired flux, further exacerbating impairment of oxygen exchange (Betticher et al., 1995). In addition, a reduction in functional capillary density and capillary recruitment under stress conditions may develop (Lipowsky et al., 1993). Using physiologically-informed numerical models, it has been confirmed that this reduced capillary recruitment is likely a consequence of: 1) a monotonic flow path taken by rigid RBC when approaching daughter capillaries, thus promoting cell transit through the least resistant pathway; 2) decreased “lingering” time for rigid RBC near bifurcations, thus reducing the probability of more distributed RBC flux, and 3) altered cell distribution per cross-sectional area of arterioles, again leading to an imbalance of cell distribution (i.e., hematocrit) downstream of bifurcations (Detterich et al., 2019; Ebrahimi and Bagchi, 2022). Figure 2 provides a partial and simplified overview of the impact that impaired cellular deformability of RBC has on flow behavior within larger vessels of the macrocirculation, and also, the flux through small microcirculatory vessels.

### Wall Shear Stress

Increased wall shear stress due to elevated apparent blood viscosity is a major stimulus for nitric oxide (NO) production by the endothelial cell nitric oxide synthase (eNOS). This effect is independent of the underlying reason for elevated viscosity (Roy et al., 2012). Emphasizing the dual role of erythrocyte deformability, rigid RBC tend to migrate towards the vessel wall thereby reducing the plasma free layer and directly increasing wall shear stress (Chen et al., 2017).

## The Role of Abnormal Hemorheological Parameters in the Pathophysiology of Various Diseases

### Clinical Consequences of Increased Blood Viscosity

Gori and Forconi proposed that hyperviscosity syndromes could be categorized based on the fact that the etiology was directly, or indirectly related to impaired rheology (Gori et al., 2007). *Primary hyperviscosity syndromes* develop as a direct result of altered rheological parameters, such as increased hematocrit or plasma viscosity, and include all forms of polycythemia, contracted plasma volume syndromes, acute/chronic leukemias, reactive leukocytosis, thrombocythemia, and platelet hyperactivity. *Secondary hyperviscosity syndromes* develop as a consequence of the adverse milieu in a variety of vascular diseases. For instance, frequent ischemic-reperfusion events are accompanied by enhanced local oxidative stress and inflammation, which in turn impact RBC deformability and aggregation. Polycythemia vera, a rare myeloproliferative disease, is characterized by increased hematocrit, rouleaux formation, blood viscosity, and thrombotic events (Kwaan and Wang, 2003). Nader reported that individuals hospitalized for COVID-19 disease exhibited increased RBC aggregation, blood viscosity, and demonstrated hypercoagulability (Nader et al., 2022). Notably, patients with the highest blood viscosity were those requiring oxygen supplementation to maintain arterial saturation within the normal range. Lamarre reported that patients with sickle cell disease and elevated blood viscosity had more frequent episodes of vaso-occlusive crises when compared to those with lower blood viscosity (Lamarre et al., 2012).

These examples clearly demonstrate that hemorheological parameters play an important role in the pathophysiology of several disorders that become apparent when the compensatory mechanisms of the body are exhausted or the vascular endothelium is compromised. For example, Intaglietta’s laboratory demonstrated in animal models that mild to moderate increases in hematocrit and, consequently, blood viscosity, may actually cause a decline in vascular resistance and blood pressure due to eNOS activation and consequent vasodilation (Martini et al., 2005). A recent study conducted in the city located at the highest elevation in the world (5,100 m above sea level, La Rinconada, Peru) reported extremely high blood viscosity values in permanent residents. In concert with extremely high hematocrit (∼65%), highlanders’ whole blood viscosity at low shear rates was almost a magnitude higher, than low-landers’ measured at sea level that presented with normal hematocrit (∼42%) (Stauffer et al., 2020). Despite the extremely elevated low-shear blood viscosity, only 25% developed related clinical symptoms such as chronic mountain sickness, suggesting well developed and highly effective vascular adaptive mechanisms in this population.

### Clinical Consequences of Increased RBC Aggregation

The impact of increased RBC aggregation on local blood flow, tissue perfusion, and vascular resistance is complex and depends on the properties of the vascular bed. RBC aggregates typically form in areas with low shear rate, such as the venous system, and rouleaux are dispersed in vascular segments with high shear rate. However, under pathological circumstances, some RBC aggregates may persist, affecting flow dynamics by promoting RBC axial migration and increasing the cell free layer width. There are multiple consequences to this: 1) decrease of apparent dynamic blood viscosity; 2) reduction in wall shear stress, which leads to lower NO production and blunted vasodilation; 3) increased plasma skimming phenomenon at bifurcations, which in turn lowers hematocrit and blood viscosity in the microcirculation (Fahraeus and Fahraeaus-Lindqvist effects), and 4) increased pre-capillary resistance owing to the persistent RBC aggregates (Cokelet and Goldsmith, 1991). Overall, predicting the clinical consequences of increased RBC aggregation *in vivo* appears to be complex. Totsimon demonstrated that increased RBC aggregation predicts mortality in patients admitted to intensive care units (Totsimon et al., 2017), while Ko suggested that it is an early indicator of sepsis (Ko et al., 2018). In sickle cell anemia, increased RBC aggregation was associated with a higher risk of developing acute chest syndrome (Lamarre et al., 2012). More recently, Nader found that the increased RBC aggregation in patients hospitalized for COVID-19 positively correlated with clot firmness, the length of hospitalization, and the extent of pulmonary lesions (Nader et al., 2022). Overall, studies have consistently documented elevated RBC aggregation in patients with cardiovascular, metabolic, and inflammatory disorders when compared to healthy individuals, and are thus likely to contribute to the adverse clinical outcomes in these populations.

### Clinical Consequences of Decreased RBC Deformability

Independent of its effect on blood viscosity, changes in RBC deformability due to a decrease in membrane elasticity, surface-to-volume ratio, or rise in internal viscosity, may have significant consequences in clinical practice (Clark et al., 1983). For example, in hereditary spherocytosis, the lack of a protein facilitating the interaction between the cytoskeleton and lipid bilayer (i.e., α-spectrin, β-spectrin, ankyrin, band 3 or protein 4.2) leads to reduced RBC deformability and increased fragility. In addition, the enhanced microparticle release into the circulation will result in a reduction to the RBC surface area, further decreasing deformability (Da Costa et al., 2001). These hemorheological alterations may contribute to the clinical signs and symptoms of hereditary spherocytosis, including hemolytic anemia, jaundice, splenomegaly, and cholelithiasis. Other inherited RBC membrane disorders, such as hereditary elliptocytosis, Southeast Asian ovalocytosis, hereditary pyropoikilocytosis, and hereditary stomatocytosis are also characterized by the presence of fragile RBC with decreased deformability (Da Costa et al., 2013). Indeed, patients with these disorders develop clinical characteristics similar to those with hereditary spherocytosis.

As detailed above, RBC deformability is highly dependent on a normal cellular surface-to-volume ratio. The internal volume of individual erythrocytes is tightly regulated by ion channels (von Lindern et al., 2022) and any change in the activity of these, such as in channelopathies, may have detrimental consequences. Hereditary xerocytosis (i.e., dehydrated stomatocytosis) is often cited as an example and is caused by a gain-of-function mutation in *PIEZ O 1* (von Lindern et al., 2022; Picard et al., 2019). Associated clinical consequences include anemia, hemolysis, and iron overload (Picard et al., 2019). Gardos channelopathy is characterized by a gain-of-function mutation in the Gardos channel (*KCNN4*). This prompts increased potassium leakage from the RBC accompanied by mild to moderate RBC dehydration. It shares clinical characteristics with hereditary xerocytosis.

Sickle cell anemia is often considered as the prototype of RBC rheological diseases. It is caused by a single nucleotide mutation (adenine → thymine) in exon I of the β-globin gene, prompting the substitution of valine for glutamic acid at the sixth position of the β-globin chain. The resultant sickle hemoglobin polymerizes when deoxygenated, leading to the mechanical distortion of RBC. Although sickling is a reversible process when hemoglobin S is reoxygenated, the repeated cycles of sickling-unsickling ultimately cause permanent damage to the RBC membrane leading to the loss of global deformability, independent of oxygenation status (Connes et al., 2016). Contributing to the pathophysiology, the deoxygenation of sickle RBC is accompanied by an increase of calcium influx through the P-sickle pathway. This activates the Gardos channel which leads to RBC dehydration and elevated internal viscosity, thus further increasing the tendency of hemoglobin S to polymerize (Brugnara, 2018). Sickle RBC are rigid and do not traverse the microcirculation easily, they are also fragile and thus prone to hemolysis. Therefore, affected patients develop several acute and chronic complications, such as episodes of painful vaso-occlusive crises, stroke, acute chest syndrome, priapism, pulmonary hypertension, leg ulcers, osteonecrosis, retinopathy, maculopathy, glomerulopathy, anemia, and vascular dysfunction.

Hemoglobin C is a common hemoglobin variant caused by the substitution of lysine for glutamic acid at the sixth position of the β-globin chain (Karna et al., 2022). RBC from individuals with homozygous hemoglobin C disease are extremely dehydrated with severely reduced deformability, which is even more profound than in sickle cell anemia (Lemonne et al., 2016). These patients not only have chronic hemolytic anemia, but the presence of very rigid RBC prompts the development of hyperviscosity (Karna et al., 2022). A small study in patients with hemoglobin C disease showed that, despite the profound hemorheological abnormalities, none of the seven individuals had any hospitalization for painful vaso-occlusive crisis or acute chest syndrome (Lemonne et al., 2016). The exact reason for this is not completely understood, but highlights the different clinical phenotype from sickle cell disease. In case of individuals with hemoglobin C disease, the very low cellular volume could potentially enable RBC to traverse through the microcirculation normally and to deliver oxygen to tissues despite the fact that deformability is severely reduced.

**FIGURE 3:**
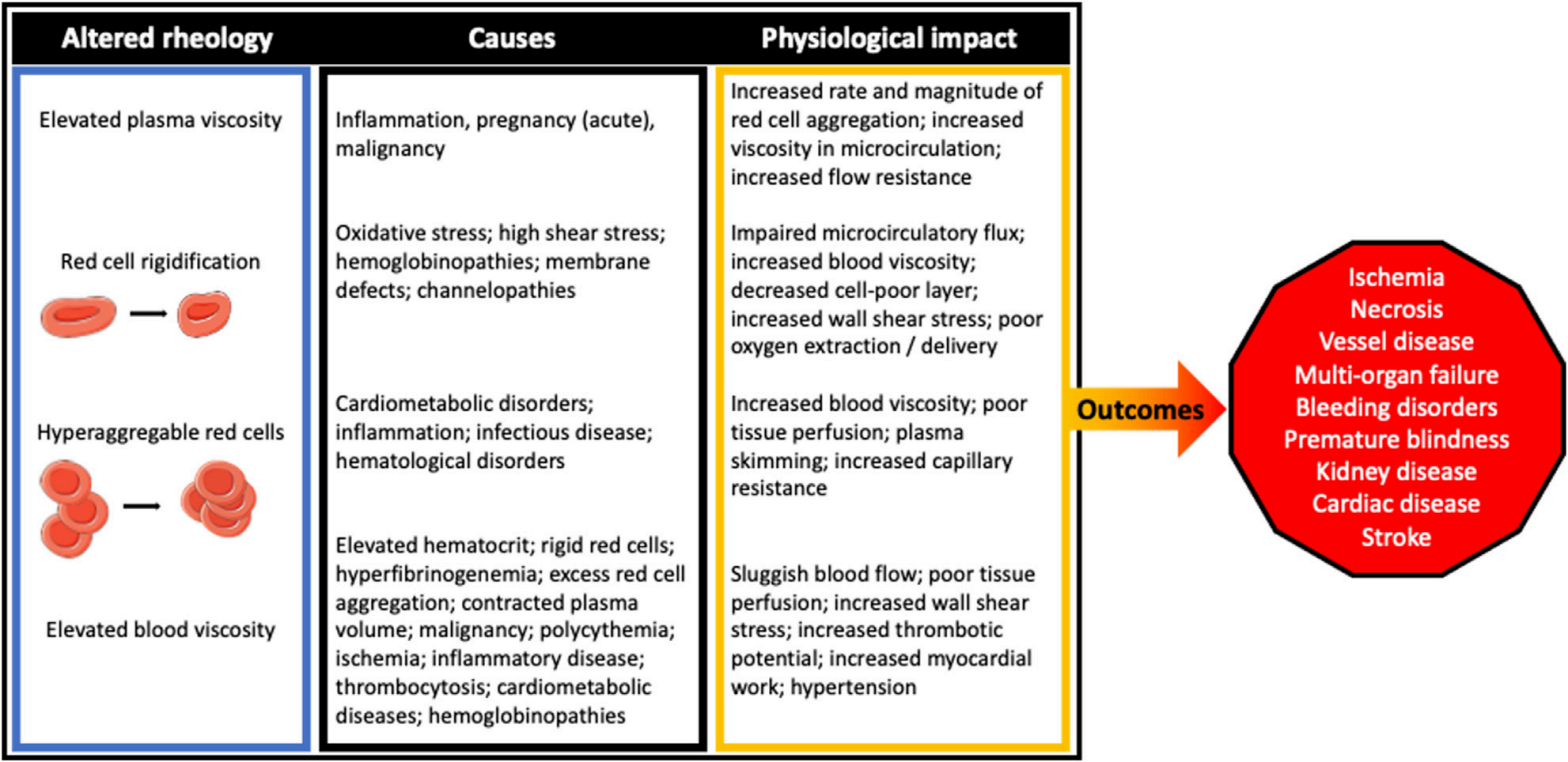
The clinical outcomes for those suffering from rheological disturbances have seemingly diverse, although clearly interconnected pathogeneses. Irrespective of the cause, the impact of perturbed blood rheological properties tend to impair blood flow at both the macro- and microcirculatory scale.

A summary of the impact that abnormal blood rheology has on cardiovascular and circulatory physiology is provided in Figure 3, with known causes and clinical outcomes also provided.

## Novel Concepts and Future Trends in Blood Rheology

### The Role of Nitric Oxide

The most widely established role of NO in the human body is to aid in regulation of vascular tone through its effect on the smooth muscle cells (Moncada et al., 1991). Evidence over the past 2 decades has, however, revealed its direct effect on RBC rheology (Simmonds et al., 2014). Bor-Kucukatay demonstrated that incubation of healthy RBC with sodium nitroprusside, a NO donor, prompted a decrease in RBC aggregation (Bor-Küçükatay et al., 2000). In contrast, administration of l-NAME, an eNOS inhibitor, led to an increase in RBC aggregation in rats. The exact underlying mechanisms to explain these findings remain unclear but might involve membrane/cytoskeletal protein nitrosylation or oxidative stress modulation. In addition to its effect on aggregation, NO also affects RBC deformability. Starzyk showed that intravenous administration of l-NAME to rats causes a reduction in RBC deformability (Starzyk et al., 1997), a finding later also reported by Bor-Kucukatay (Bor-Kucukatay et al., 2003). Nader demonstrated that incubation of sickle RBC with sodium nitroprusside mildly increased cellular deformability, decreased the percentage of RBC with externalized phosphatidylserine, but had no effect on microparticle release (Nader et al., 2020). Overall, these findings suggest that external administration of NO may favorably modulate the rheological properties of RBC, but the achieved improvements in patients with severe abnormalities are likely limited.

Kleinbongard was the first to demonstrate that RBC express a functional eNOS-like enzyme (RBC NOS), which is localized to the membrane but also found in the cytoplasm (Kleinbongard et al., 2006). Inhibition of RBC NOS led to a concomitant decline in RBC deformability. Grau extended these findings showing that RBC NOS activation by insulin leads to increased NO content of RBC and improved cellular deformability through direct S-nitrosylation of cytoskeletal proteins (Grau et al., 2013). In contrast, inhibitors of the same enzyme (wortmannin or L-N5-(1-Iminoethyl)-ornithin) prompted a decrease in RBC NO content, cytoskeleton protein S-nitrosylation, and RBC deformability. In a follow-up *in vitro* study, Grau exposed RBC from controls and patients with sickle cell disease to shear stress, which led to an increase in RBC NOS activity in both populations but deformability was only impacted in cells from healthy individuals (Grau et al., 2015). It was also demonstrated that this intracellular pool of NO generated by shear may diminish the impact of oxidative stress, at least in *ex vivo* models (Kuck et al., 2019). Ulker found that the shear stress-mediated influx of extracellular calcium is a prerequisite for RBC NO generation, and through the use of NOS inhibition, provided indirect evidence that RBC NOS activation may also be dependent upon calcium flux (Ulker et al., 2018), an effect that was recently demonstrated to be dependent upon Piezo1 activation via mechanical stimulation to the RBC membrane (Kuck et al., 2022).

It is important to mention that RBC release adenosine triphosphate (ATP) in response to hypoxia or deformation caused by mechanical stress. ATP binds to purinergic receptors on the surface of endothelial cells leading to eNOS activation and NO release. The generated NO may reach the vascular smooth muscle cell layer, thus inducing vasodilatation and enhanced microvascular blood flow (Ulker et al., 2018). Because RBC deformation is an important stimulus for ATP release from RBC, decreased deformability, including aging, may lead to increased vascular resistance (Price et al., 2004).

### RBC Rheology and Hemolysis

Severe reduction in RBC deformability, as observed in hemoglobinopathies, results in a high hemolysis rate, even at physiological shear forces (Connes et al., 2014). Hemolysis has been shown to also occur in the setting of very high shear stress exposure, even in healthy cells (Zhang et al., 2011). In addition, it has been demonstrated that accumulated shear history, even to non-hemolytic levels, reduces the tolerance of RBC to subsequent shear (Baskurt and Meiselman, 2013). A loss of RBC deformability due to supraphysiological, but sub-hemolytic shear stresses occurs prior to overt hemolysis (Horobin et al., 2017). Collectively, these data support that the mechanical properties of RBC are vital to cell survival, and once perturbed, hemolysis rapidly ensues.

Accumulating data demonstrates that hemolysis may lead to vascular dysfunction through different mechanisms: 1) released plasma free hemoglobin reacts with NO to form methemoglobin and nitrate, which are unable to contribute to vasomotor tone regulation (Kato et al., 2007; Detterich et al., 2015); 2) the release of intracellular arginase leads to l-arginine consumption which serves as a NO precursor; 3) asymmetric dimethylarginine (ADMA), an endogenous NOS inhibitor abundant in RBC, is released during hemolysis (Davids et al., 2012). In addition, the release of ADMA and the depletion of l-arginine provoke NOS uncoupling, increasing the production of reactive oxygen species (Antoniades et al., 2009). Plasma free-hemoglobin can also directly activate innate immune pathways through TLR4 and NLRP3 inflammasome signaling, promoting chronic inflammation (Conran and Belcher, 2018). The release of IL-1β will increase and bind to the IL-1 receptor on leukocytes and endothelial cells. This initiates a cascade of events that ultimately activate neutrophils, platelets and upregulate E-selectin, P-selectin, VCAM-1, ICAM-1, and cytokine expression in endothelial cells (Kato et al., 2017). Detailed overview of these regulatory pathways and mechanisms are beyond the scope of this review; however, their importance is emphasized by the recent development of novel therapies aimed at limiting the detrimental effects of hemolysis, such as hemopexin, voxelotor, or the use of haptoglobin.

### RBC Senescence and Vascular Function

RBC have an average life span of 120 days in the human circulation, after which they are cleared by reticuloendothelial macrophages. RBC aging is accompanied by a reduction in deformability due to several factors: 1) cell shrinking; 2) band 3 clustering mediated by the accumulation of oxidized hemichrome (Low et al., 1985); 3) band 4.1 deamidation leading to two distinct forms of band 4.1 with different molecular weights (4.1a and 4.1b) (Mueller et al., 1987); 4) loss or conformational changes of CD47 (Burger et al., 2012), and 5) increased externalization of phosphatidylserine to the RBC membrane surface (Lang et al., 2008). This has been shown to promote the adhesion of RBC to the endothelium in several diseases associated with vascular injury, such as retinal vein occlusion, chronic uremia, and sickle cell disease. Parallel with these processes, microvesicles are released from the RBC throughout their life span (Leal et al., 2018). Vesiculation occurs to eliminate the accumulated harmful components described above to prolong as much as possible the life span of RBC. Indeed, extracellular vesicles (i.e., microparticles or microvesicles) are generated during normal aging of RBC, by plasma membrane budding (Leal et al., 2018). Increased number of RBC-derived microparticles have been demonstrated in the plasma of patients with various diseases that are associated with high oxidative stress and inflammation. Conversely, plasma accumulation of microparticles can potentially promote the development and progression of vascular disorders. Increased concentrations of circulating RBC-derived microparticles have been reported in sickle cell disease patients with pulmonary hypertension, acute chest syndrome, and stroke, as well as those who had a history of thrombosis or splenectomy (Tantawy et al., 2013). A positive correlation has been observed between arterial rigidity and the plasma level of RBC-derived microparticles in the same population (Nader et al., 2020). It was demonstrated that these exert a deleterious effect on endothelial cells through TLR4 activation, inducing ICAM-1 and E-Selectin expression, and cytokine production.

## RBC Rheology and Coagulation

It is generally believed that RBC play a passive or only minor role in thrombosis and hemostasis. However, recent studies have demonstrated that they have biologically and clinically important functions in hemostasis and coagulation disorders (Weisel and Litvinov, 2019). As discussed previously, phosphatidylserine is externalized to the RBC surface during its normal aging process which, in turn, promotes thrombin generation. Under certain conditions, this may account for up to 40% of prothrombin activation (Whelihan et al., 2012). Phosphatidylserine-rich microparticles released from aging or damaged erythrocytes also contribute to this process (Morel et al., 2011) and may serve as targets for novel antithrombotic therapies.

Clot contraction is an important step in achieving effective hemostasis. It is driven primarily by platelet contraction which is accompanied by RBC compression and shape change to polyhedral (polyhedrocytes) (Cines et al., 2014). Tutwiler and Faes have shown that sickle and artificially rigidified RBC are unable to form polyhedrocytes, thereby impacting clot structure, and clot retraction (Faes et al., 2019; Tutwiler et al., 2021). These findings emphasize the role of RBC rheology in the normal process of hemostasis. Further studies are needed to evaluate if these observations would offer a potential for therapeutic interventions.

## The Role of Hemorheology in Mechanical Circulatory Support Devices

Mechanical circulatory support (MCS) device use continues to increase in patients with refractory cardiogenic shock and end stage heart failure. With a few exceptions, such as the intraaortic balloon pump that will not be considered here, a rotary pump with an impeller providing continuous blood flow is a critical component of these systems. MCS devices may be categorized based on a number of criteria: 1) level of support provided (complete or partial); 2) the ventricle supported (left, right, or biventricular); 3) implantation in the body (intracorporeal or extracorporeal); 4) insertion technique required (surgical or percutaneous); 5) intended duration of use (temporary or durable), and 6) pump design based on flow pattern (axial or centrifugal). Thorough knowledge of these devices is critical to ensure the best pump is selected to match the demands of the clinical scenario. Furthermore, understanding their hemocompatibility and the effect of hemorheological variables on their efficacy will reduce complications.

### The Role of Hemorheology in MCS Performance

Hemorheological parameters, such as plasma viscosity, hematocrit, and blood viscosity are critical determinants of MCS device hydrodynamic performance. These directly affect the density of blood and therefore the head pressure, hydraulic power, and efficiency of the pump. Given the exponential relationship between hematocrit and blood viscosity, it is important to maintain hematocrit in the low-normal range. While higher hematocrit may support enhanced oxygen transport, it also has a profound impact on blood viscosity and thereby, resistance to flow. Thus, a careful balance of these competing factors should promote optimal convective transport. Elevated blood viscosity also increases the risk of blood stagnation within the pump housing and consequently, thrombus formation.

### Hemorheology, Hemocompatibility, and MCS Devices

Hemocompatibility is a term that incorporates the hemolytic tendencies and the thrombogenic potential of a given medical device. It is important to consider that several factors influence hemocompatibility, including the device material, surface roughness, flow path, and shear forces that exist within a given device. In case of suboptimal interactions of these determinants with blood, hemolysis and/or activation of the coagulation cascade is likely (Hilal et al., 2019). With regards to MCS devices, hemocompatibility-related adverse events include, but are not limited to thrombosis, stroke, gastrointestinal bleeding, and hemolysis. In addition, the role of hemorheological parameters is especially important when considering MCS support systems.

The Impella family of devices (2.0, CP, 5.0 and 5.5; Abiomed, Danvers, MA) are inserted across the aortic valve and into the left ventricle to support a failing heart. Due to the small size of the impeller (12–14 French, or ∼4 mm outer diameter), its rotation exceeds 40,000 revolutions per minute (RPM) substantially increasing the risk of shear stress-induced hemolysis (Badiye et al., 2016). In addition, the relatively high flow rates (up to 5.5 L/min) through a small inner diameter flow path invariably lead to high levels of blood trauma (Sibbald and Džavík, 2012). Thrombotic complications are relatively infrequent owing to the purge fluid which contains heparin most commonly. Veno-arterial extracorporeal membrane oxygenation (VA-ECMO) systems use two cannulas to drain and re-inject large volumes of blood to provide oxygenation as well as full hemodynamic support to both ventricles. The role of blood viscosity is critically important in these systems given the high volumetric minute-flow and the lengthy tubing system required between the patient and the extracorporeal centrifugal pump. The use of a larger diameter impeller allows for lower rotational speed, thus reducing the risk of hemolysis.

Durable left ventricular assist devices (LVAD) are increasingly used for patients with end stage heart failure. They are surgically implanted given the intent for long-term life-support and their prospects are highly dependent on outstanding hemocompatibility. The Heartmate II (HM-II; Abbott Laboratories, Minneapolis, MN) is a magnetically driven, compact, axial flow pump, meaning that its rotor and bearing align with the direction of flow. By using a smaller diameter impeller, it operates within a high-speed range, typically between 8,000 and 12,000 RPM, which leads to high shear forces and thus increased risk of damage to the cellular elements of blood. Significant acute hemolysis may have immediate profound adverse effects on end organ function, such as promoting pigment-induced nephropathy and renal failure. In addition, plasma free-hemoglobin may be catabolized into carbon monoxide which increases the velocity of thrombus growth, thrombus strength, promotes hyperfibrinogenemia, and impairs fibrinolysis (Sibbald and Džavík, 2012). Related to the fast rotational speed, the excess heat generated needs to be dissipated by the volumetric blood flow in order to avoid localized coagulation and thrombus development. The regions along the diffuser guide vanes and the ruby bearings used for impeller suspension are especially prone to clot formation in the HM-II device. The high shear rate in the vicinity of the impeller also has a profound effect on von Willebrand Factor (vWF); specifically, when shears exceed 5000 s^−1^, the structure of vWF is altered such that critical domains are exposed which accelerates enzymatic degradation of this protein (Hilal et al., 2019). This leads to acquired von Willebrand disease (avWD), similar to that described in severe aortic stenosis (Heyde syndrome). The onset of avWD is almost instantaneous following LVAD surgery. The breakdown of vWF is further facilitated by the enzyme ADAMTS13, ultimately increasing the risk of hemorrhagic complications (Selgrade and Truskey, 2012; Bartoli et al., 2017). It is important to note that the degradation of high molecular-weight vWF multimers are most effective at providing hemostatic regulation; shear induced reduction in vWF molecular weight and/or transition of multimers towards monomers of this very large protein results in poor hemostatic potential.

The newer generation LVADs, such as the HeartWare (HVAD; Medtronic Inc, Minneapolis, MN; withdrawn from the market in 2021) and the HeartMate III (HM-III; Abbott Laboratories, Minneapolis, MN) use centrifugal pump design; that is, rather than employing an axial flow design, these pumps provide flow in a radial manner. A series of blades are integrated into a disc-shaped impeller, which is magnetically levitated and/or employs a hydrodynamic bearing, thereby eliminating friction and heat generation. Blood flows into the eye of the impeller via a suction inlet, then is accelerated by the centrifugal force pushing it towards the spiral-shaped device housing and ultimately through a discharge outlet. This is connected to the ascending aorta by a flexible outflow graft. By design, centrifugal pumps use impellers with a larger diameter and thus allow for lower rotational speeds. This increases their reliable lifetime, lowers local heat generation, and reduces the shear forces exerted on blood cells thereby decreasing the risk of hemolysis and improving hemocompatibility. As an example, the centrifugal-flow design EVAHEART LVAS (Evaheart, Houston,TX) was compared to the HM-II, and it was found that the EVAHEART LVAS exerted less than half of the shear forces on the blood components, when compared to the HM-II. A marked reduction in vWF cleavage was observed in the EVAHEART LVAS, leading to lower risk of hemolysis and thrombus formation (Bartoli et al., 2017). The HM-III device was also designed with larger gaps between the impeller and the device housing, further reducing shear forces, blood trauma, and the risk of thrombus formation. Geisen and others compared the prevalence of avWF disease in patients with HM-II with that in HM-III, and found that this complication rate was significantly lower in patients with the HM-III system which is likely responsible, at least in part, for the fewer documented bleeding events (Geisen et al., 2018).

Hemolysis is a common phenomenon following LVAD implantation. Despite the significant advances in MCS technology, the laboratory test we clinically utilize for early hemolysis detection remains serial plasma lactate dehydrogenase (LDH) level monitoring. LDH concentration exceeding 2.5 times the upper limit of normal may be of diagnostic value. In addition, plasma free-hemoglobin concentrations may exceed 40 mg/dl and haptoglobin levels are typically low or undetectable (Katz et al., 2015; Akin et al., 2016; Hilal et al., 2019). Often late and less sensitive indices for hemolysis include elevated pump power consumption, abnormally high *reported* pump flow, increased pulsatility index, or the development of new heart failure symptoms (Katz et al., 2015; Akin et al., 2016).

### Clinical Implications of Poor MCS Hemocompatibility

It is important to note that such negative impacts on hemolysis are associated with poor circulatory health. Sansone explored the potential impact of LVAD devices on microvascular and macrovascular function (Sansone et al., 2015), and demonstrated curious results indicating that LVAD recipients (compared with coronary artery disease, and heart failure): 1) presented with improved microvascular flux, as indicated using laser doppler techniques, although; 2) these same patients presented with significantly impaired macrovascular function as determined by flow-mediated dilation. These data support prior findings by Lou showing that axial flow LVAD devices negatively impact reactive hyperaemia, a measure of vascular function (Lou et al., 2012). Potentially shedding light on this observation, Sansone and others demonstrated a significant relation between the degree of impaired flow mediated dilation and the level of plasma free-hemoglobin, ostensibly indicating that NO bioavailability (and thus vascular function) may have been impaired secondary to hemolysis (Sansone et al., 2015).

### Impact of Shear Stress on MCS Hemocompatibility

The mechanisms that underpin blood damage in medical devices, and particularly modern mechanical circulatory support, can largely be related to the mechanical forces exerted on blood. While it is acknowledged that turbulent stresses contribute to blood damage, there is difficulty in creating reproducible models with discrete control over such flows; consequently, there is considerable research devoted to understanding the manner in which laminar shear stress induces blood damage. While blood was previously thought to be able to withstand shear stress until the so-called hemolytic threshold, which is both magnitude and duration dependent (Zhang et al., 2011), recent advancements demonstrate that hemolysis can be induced by shear stresses that are well-below the hemolytic threshold, at least in case of repetitive shear exposures. Baskurt demonstrated that the hemolytic threshold could be reduced to ∼150 Pa for blood that had been previously “conditioned” with high shear stress (Baskurt and Meiselman, 2013). Horobin and others demonstrated that even a single second of exposure to 100 Pa per minute resulted in: 1) an initial decrease in cellular deformability for 15–30 min, before; 2) hemolysis could be detected after 45–60 min (Horobin et al., 2017). These data suggested that the cellular mechanics of RBC were sensitive to shear, and with repeated exposures typical of MCS devices, these properties would gradually decline prior to overt cell rupture. Simmonds and Meiselman performed the first comprehensive mapping of RBC sensitivity to shear, and demonstrated that RBC deformability was degraded within seconds of exposure to 64 Pa, or within minutes of exposure to 38 Pa of shear stress (Simmonds and Meiselman, 2016). O’Rear found that such shear exposure leads to an impaired capacity for RBC to regulate calcium flux (O'Rear et al., 1982), which may ultimately facilitate dehydration of the erythrocyte owing to an impaired Gardos effect (Kuck et al., 2020). Kameneva demonstrated that RBC aggregation, too, was impacted by shear typical of MCS devices (Kameneva et al., 1995), which was later demonstrated by McNamee to be the result of cleavage of sialic acid from the cell membrane (McNamee et al., 2018). As discussed above, these nine-carbon atom sugars are the primary contributors to the negative electrostatic charge of RBC, and thus cleavage from the membrane promotes altered RBC aggregation. It thus appears that sublethal changes to the RBC, such as impaired cellular deformability secondary to disrupted hydration status and electrolyte handling, and impaired cellular aggregation secondary to remodeled surface chemistry, are early signs of cell damage that ultimately precipitates hemolysis.

## Conclusion

Although the flow properties of blood have been under examination for centuries, the field of hemorheology continues to evolve. Following the initial macroscopic and microscopic observations focusing on red blood cell aggregation and deformability, with the discovery of NO and its critical multi-faceted role, the field continues to expand towards molecular and biochemical mechanisms. An increasing number of cardiovascular and hematological disorders have been linked to abnormal hemorheological parameters enabling the potential development of novel therapeutic interventions. In addition, optimizing shear forces, flow patterns, and hemocompatibility in temporary and durable mechanical circulatory support devices is of critical importance with an immediate effect on patient survival. With interested researchers and clinicians joining the field, the possibilities for meaningful discoveries will continue to grow and evolve.
